# Building community capacity for sustainable local food systems in central Java, Indonesia: an integrated training evaluation framework

**DOI:** 10.3389/fnut.2026.1939048

**Published:** 2026-10-14

**Authors:** Agus Subhan Prasetyo, Kim E. Dooley

**Affiliations:** 1 Department of Agricultural Leadership, Education, and Communications, Texas A&M University, College Station, TX, United States; 2 Department of Agriculture, Faculty of Animal and Agricultural Sciences, Universitas Diponegoro, Semarang, Indonesia

**Keywords:** ADDIE, capacity building, food security, kirkpatrick evaluation, local food processing, sustainable food systems

## Abstract

Transitioning to a safe, resilient and sustainable food system requires more than policies and local food resources; it requires building community capacity to convert that potential into sustainable methods. However, there is little evidence of training's contribution to local food systems beyond the immediate learning outcomes. This study explores the contribution of a local food processing training program to community capacity building and sustainable local food systems through an integrated evaluation of its program design, implementation, and outcomes. We performed a qualitative case study with multiple perspectives of the Local Food Processing Training Program (LFPTP) organized by the Food Security Agency of Central Java Province, Indonesia. Data were collected through program documents, semi-structured interviews with trainers, and in-depth interviews sessions with 15 participants. Data were analyzed using thematic analysis and cross-perspective synthesis using the ADDIE model, the Kirkpatrick Evaluation Model and the FAO Four Pillars of Food Security. The results point to two main mechanisms affecting the effectiveness of the program. First, there is a “design-implementation gap” when trainers must adapt the curriculum to the diversity of participants' experiences and different constraints in implementation. Second, there is a “transfer gap” where knowledge and skill gains do not reliably translate into changes in behavior, sustainable entrepreneurial activities or broader economic outcomes. The success of the transfer also depends on previous experience, access to resources, family support, market opportunities and local socio-economic conditions. This research adds to the extant literature by constructing an integrated evaluation framework to link instructional design, training evaluation, and food system perspectives. The framework shows that the contribution of training to sustainable local food systems depends on the inter-connectedness of design, adaptive implementation, learning transfer and contextual conditions that enable long-term application.

## 1 Introduction

Building safe, resilient, and sustainable food systems is a major global challenge. Climate change, environmental degradation, disruptions to food supply chains, population growth and changing consumption patterns have increased the vulnerability of food systems across countries. These challenges affect not only the availability of food but also the ability of food systems to deliver safe, nutritious, affordable and sustainable food in the long term (1, 2). Food security is considered not only in terms of food production but also in relation to the food system's capacity to adapt to change, and to ensure sustainability through resource management, governance, and the capacity of the communities involved (3).

Another approach that is increasingly gaining attention in support of food security is the empowerment of local food systems. This approach has been planned in several countries, because this approach considered to contribute to dietary diversification and improved public nutrition, and to strengthening of local food value chains, adding value to agricultural products, boosting local economies and increasing community resilience to economic and environmental pressures by utilizing local raw materials (3, 4).

Food-system transformation is discussed at the global and national levels, but it is implemented through actions and decisions at the local level. Households, producers, processors, community organizations, extension actors and local governments are important factors in determining how food resources are produced, processed, accessed and used. Thus, local food systems are an important entry point for improving resilience and sustainability. In this respect, developing strong local food systems requires not only the availability of local resources and enabling policies but also the knowledge, skills and capacity of local actors to translate available resources into sustainable methods and economic activities.

This need is particularly acute in Indonesia where local governments have introduced programs to promote local food, food processing and community economic development. One such public intervention was the Local Food Processing Training Program held by the provincial Food Security Agency in Central Java. The program was intended to strengthen community capacity to process locally available food resources and encourage value-added, local food-based economic activities. Training therefore served as one mechanism through which broader food-security and local-development objectives could be translated into practices at the community level. However, the contribution of training to food-system transformation cannot be assumed simply from the delivery of training activities or improvements in participants' immediate learning outcomes. The extent to which training can lead to longer-term change depends on the quality of the program design, how it is adapted during implementation, and whether participants are able and supported to transfer what they learn to practice.

Prior research on training has shown that learning outcomes do not necessarily lead to sustained behavioral or organizational change. Training transfer is affected by individual differences, training context, and post-training support and resources (5–7). This problem is especially relevant when dealing with extension and capacity building for agriculture and food, where participants generally operate in different social, economic, institutional and market environments. Thus, to understand the contribution of training, one must consider not only whether participants learned, but also the design and implementation of training, and the application of learning within the local food system.

These processes can be examined from several complementary conceptual perspectives. The ADDIE model provides a systematic instructional-design process that consists of five phases of analysis, design, development, implementation, and evaluation (8, 9). It is primarily focused on the study of the planning, development, delivery and refinement of learning interventions. Conversely, the Kirkpatrick Evaluation Model is an evaluation model focused on the outcomes of training and has four levels: Reaction, Learning, Behavior and Result. This is particularly useful to explore if training experiences and learning outcomes cause changes in practice and broader results (10, 11). The FAO Four Pillars of Food Security (availability, access, utilization and stability) provide a food-system lens to understand the broader meaning of these changes (1, 3). Thus, each framework addresses a different part of the evaluative problem: ADDIE helps explain how the intervention was designed and implemented, Kirkpatrick examines what changed through the training, and the FAO framework provides a basis for considering what these changes may mean for food security and local food systems. Integrating the three frameworks creates an analytical pathway linking training design and implementation, learning, earning transfer and contribution to local food-system capacity. This integration is particularly relevant for public agricultural and food-extension programs in which educational interventions are expected to contribute to broader social and economic objectives.

This study forms part of a broader research series examining the same Local Food Processing Training Program. An earlier study by Prasetyo et al. (12) examined the Analysis and Design phases of the ADDIE model using program documents, focusing on the program's instructional design and its alignment with the FAO food-security pillars. The present study builds on that work but addresses a broader and distinct analytical question. Rather than focusing only on the initial design stages, it synthesizes evidence across the training cycle by incorporating trainer perspectives on Development and Implementation and participant experiences of training outcomes and post-training application. Thus, although the studies share the same program context and documentary evidence from the initial stages, the present study extends the analysis through cross-perspective synthesis to examine how design, adaptive implementation, learning transfer, and contextual conditions interact.

Accordingly, this study aimed to develop a more comprehensive understanding of how local food processing training contributed to community capacity building and sustainable local food systems. The research combined the ADDIE model, the Kirkpatrick Evaluation Model and the FAO Four Pillars of Food Security to investigate the connections between program design, implementation, learning, transfer of training and the contribution of participants to local food-based entrepreneurship and food-system capacity. Materials and Methods

### 1.1 Study design

A qualitative case study design with a multi-perspective evaluation approach was used to explore the contribution of a local food processing training program to community capacity building, local food-based entrepreneurship, and the strengthening of local food systems. The approach was selected because it enabled the examination of the relationships among program design, training delivery, participants' experiences, and post-training outcomes in close detail within a real-world setting (13).

Typically, training evaluations use a single source of evidence to determine program effectiveness. However, this study employed multiple perspectives to develop a more holistic understanding of the processes linking instructional design, training implementation, learning transfer, and subsequent participant changes. Therefore, the study examined not only the effectiveness of the training program, but also the contribution of the learning process to building community capacity within the larger context of strengthening sustainable local food systems.

This study used the case of the Local Food Processing Training Program of the Food Security Agency of Central Java Province, Indonesia, in 2024. This program is a form of local government intervention, targeting Micro, Small, and Medium Enterprises (MSMEs) engaged in processing local resource-based food processing activities, particularly cassava. The training aims to improve participants' knowledge and skills in value-adding local food ingredients into products, while also promoting the use of local food and development of food-based economic activities at the community level. Thus, the program targets not only the improvement of technical food processing skills but also the strengthening of community capacity, diversification of local food utilization, and the promotion of food-based entrepreneurship. The program delivery comprised material presentation, demonstrations, hands-on practice, discussions, and mentoring, spanning five sessions over the course of a year.

The context of study was selected as Central Java because this program is an example of an intervention by local government that directly links community capacity building with the utilization of local food resources. The selection of this case also allows the research to move beyond the evaluation of the program planning stage and to explore how the devised design was implemented in training, how trainers adapted implementation to the circumstances of the participants and the degree to which learning outcomes were subsequently applied by participants after the training. Thus, this case is a suitable context to study the entire training process, from the design to the transfer of learning and its contribution to the local food system.

### 1.2 Conceptual framework

This study was guided by three complementary conceptual frameworks. The ADDIE model was used to explore the training design and implementation process in five key stages; Analysis, Design, Development, Implementation and Evaluation (8). The Kirkpatrick model was used to evaluate the results of the training at four levels Reaction, Learning, Behavior, and Results (10). The contribution of the training to sustainable local food systems was interpreted using the FAO Four Pillars of Food Security framework (availability, access, utilization and stability) (1).

The three frameworks are best thought of as complementary analytical lenses, not as individual models. ADDIE is mostly used to track the process of identifying program needs, designing and developing training and translating design into implementation. The Kirkpatrick model is used to understand the training experience and outcomes from the perspective of the participants. It ranges from the initial reaction to the training, to the actual learning, the application of the skills and the results after the training. At the same time, these results are placed within the wider food system through the FAO's Four Pillars framework, specifically their connections to food availability, access, utilization and stability.

We incorporate these three frameworks incrementally during the analysis. First, data from each source are analyzed to identify codes and themes that emerge from empirical experiences. These codes are then mapped and interpreted using relevant constructs from ADDIE, Kirkpatrick, and the FAO's Four Pillars. This process allows a single empirical finding to be understood from multiple perspectives where clear conceptual links exist. For instance, a trainer's adaptation of training materials is analyzed in relation to the Development and Implementation stages of ADDIE. When such adaptations relate to participants' experiences and understanding, the same finding is then compared against the Reaction and Learning dimensions of the Kirkpatrick model. Furthermore, when skills acquired by participants are applied to utilize local food ingredients or develop food-based economic activities, the implications are interpreted through the Utilization and Access dimensions of the FAO framework.

Thus, integration is not achieved by forcing all data into predetermined categories. Instead, the three frameworks help interpret patterns emerging from the data and explain the interrelationships between processes. The synthesis links program design, adaptive implementation, learning, transfer of learning, and contributions to the local food system and considers contextual factors affecting the application of training outcomes. The conceptual structure is the basis for the development of the empirical evaluation framework that has emerged from the research. The conceptual framework is illustrated in Figure 1.

**Figure 1:**
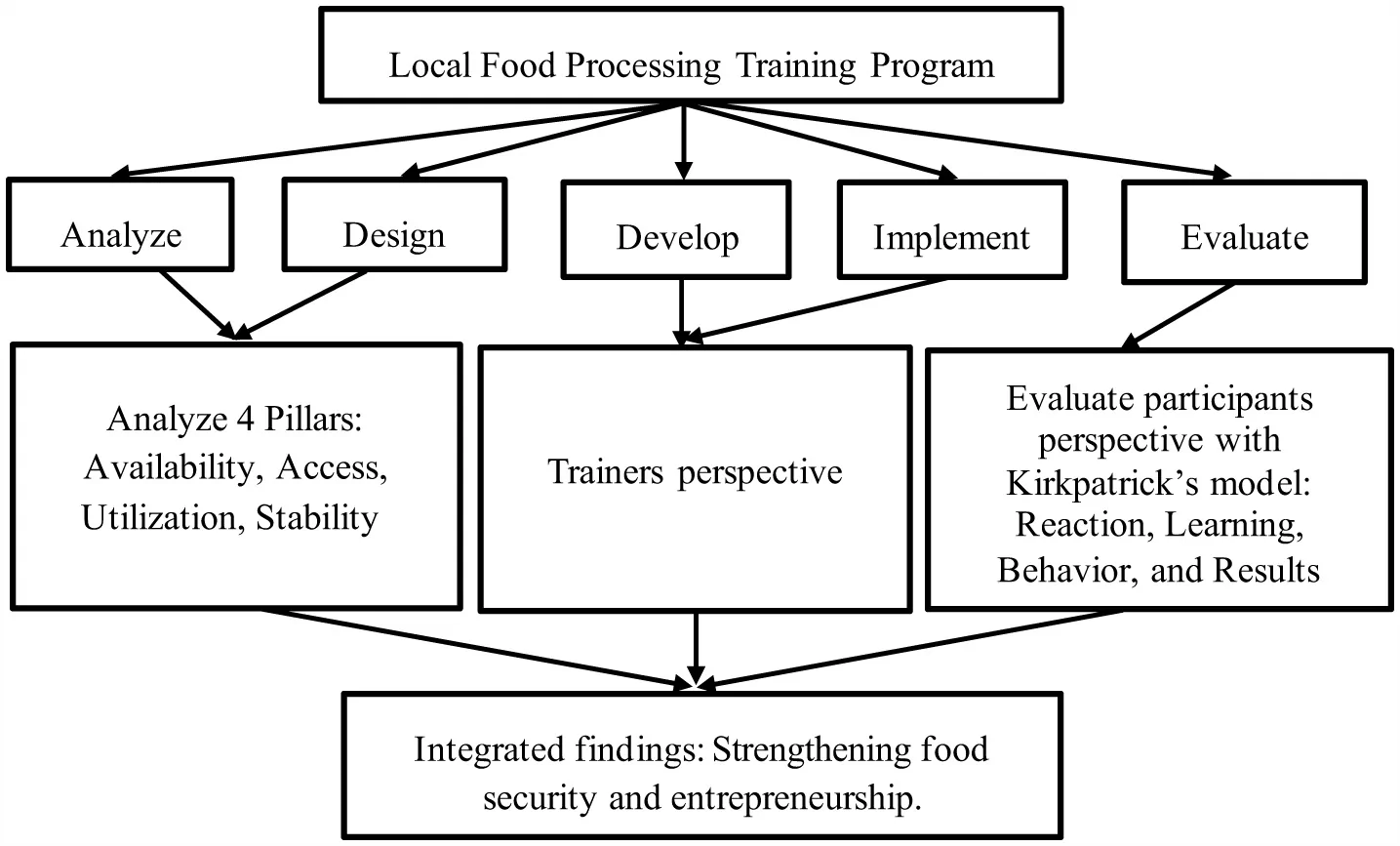
Conceptual framework for the food processing training program.

### 1.3 Data sources and participants

Three complementary sources of evidence were used to develop a comprehensive understanding of the training program (13). First, 7 program documents, including 5 training modules and 2 the final training report, were analyzed to examine how training needs were identified, how the program was designed, and how learning objectives were aligned with local food utilization. Second, semi-structured interviews were conducted with 5 trainers involved in implementing the program. A census approach was used, including all trainers in the study. Their perspectives illuminated how the program design was operationalized in instructional practice, the adaptive strategies employed in training delivery, and the issues encountered during implementation. Third, the selection of training participants was conducted purposively based on their direct and sustained involvement in the program. Specifically, the study involved 15 participants who had completed the entire series of five training sessions organized as part of the program. This criterion was deemed essential for evaluating the transfer gap because participants who attended the full series had adequate exposure to the training materials, learning processes, and program implementation; this allowed them to provide a more comprehensive account of what they had learned and the extent to which that learning was applied in practice after the training. Thus, completing all sessions served not only as an inclusion criterion but also enabled the research to explore the relationship between learning outcomes and their application in practice (behavior), the primary focus in identifying the transfer gap. The 15 participants also reflected diversity in terms of prior experience, business characteristics, and local food products, thereby allowing the study to capture various ways in which training outcomes were applied in practice (see Table 1).

**Table 1:** Characteristics of training participants.

Participant characteristic	Category	*n*
Previous experience	Less than 5 years	2
	5–10 years	6
	More than 10 years	7
Business size	Micro	2
	Small	9
	Medium	4
Local food products	Mocaf	5
	Bakery products	8
	Catering services	2
Total		15

Data adequacy was considered iteratively throughout the data collection and analysis process. Interviews were conducted and analyzed in stages, with attention paid to whether subsequent interviews yielded new codes, meanings, or patterns relevant to the research questions. With the development of the analysis, the interviews conducted in the final stages increasingly supported previously identified patterns, while also showing variations in participant experiences. Thus, the current study defined data adequacy by the depth, relevance, and sufficiency of the data gathered to explain the learning process and its post-training application, rather than defining data saturation in terms of the number of participants. Thus, the sample of 15 participants was deemed adequate to generate rich data for reflexive thematic analysis to identify variations and patterns in the transfer gap.

This sample was not intended to be statistically representative of all training participants or the whole community of Central Java. Information-rich cases of individuals with direct and comprehensive experience with the program were gathered using a purposive sampling approach. Participants were chosen for their full engagement in the program and diversity of experiences and business contexts. This allowed the research to not only assess whether participants gained knowledge from the training but to explore the conditions that fostered or inhibited the use of this learning in practice. What this meant was that the success of a training program was not just in how the participants reacted to the training or how much they learned during the training, but also in how the learned information was transferred into actual behavior and practice after the training.

### 1.4 Data collection

Data were collected from November 2025 to February 2026. Program documents were obtained from the Food Security Agency to identify the program's objectives, implementation strategies, instructional materials, and approach to local food utilization and food security. Trainers were interviewed online in semi-structured interviews lasting about 45–60 min. The interview questions focused on developing instructional materials, implementing instruction, challenges encountered during training delivery, and changes made during the learning process. Moreover, 15 training participants were interviewed in depth to explore their experiences before, during, and after the training. Interviews continued until the accounts provided sufficient depth to address the research questions and additional interviews no longer substantially expanded the emerging themes concerning participants' training experiences, learning, application of skills, and post-training outcomes.

The study was approved by the Texas A&M University Institutional Review Board (IRB) via an Exempt Determination prior to the start of data collection. All participants received an information sheet about the study and provided informed consent prior to taking part in the interviews.

### 1.5 Data analysis

This approach was selected because it enables an in-depth interpretation of participants' experiences and offers flexibility in integrating diverse sources of qualitative evidence. Analysis was conducted iteratively across three data sources: program documents, interviews with trainers, and interviews with participants. To minimize potential bias and enhance the credibility of the interpretation, the researchers compared findings from the three sources through data source triangulation, reflexively reviewed assumptions and analytical decisions during the coding and theme development processes, and documented the analytical process and decisions to create an audit trail. Thus, the manual analysis was carried out through a systematic, transparent, and reflective process, rather than relying solely on the researcher's spontaneous interpretation.

The first stage began with familiarization with the entire dataset through repeated readings of program documents, interview transcripts, field notes, and narratives. Subsequently, initial coding was performed manually to identify data segments relevant to the research questions. At this stage, codes were developed based on the content and meaning of the data, rather than solely on categories predetermined by the three conceptual frameworks.

In the next stage, initial codes were grouped into broader themes and interpreted using the three frameworks as analytical lenses. Codes related to needs assessment, design, material development, and training delivery were mapped to the relevant ADDIE stages. Codes describing participant experiences, gains in knowledge and skills, application of learning outcomes, and post-training results were interpreted using the four levels of the Kirkpatrick Model. Meanwhile, findings indicating relationships between training outcomes and food resource utilization, economic activities, and conditions supporting food system sustainability were interpreted through the dimensions of the FAO's Four Pillars of Food Security.

This mapping process was carried out flexibly. A single data segment could relate to more than one construct when a conceptual link was supported by empirical evidence. For instance, when a trainer explained that training materials needed to be tailored to participants' experiences, the finding was initially understood as a form of adaptation during the implementation process. This finding was then analyzed in relation to the Development and Implementation stages of ADDIE. If participant experiences indicated that such adaptation helped them understand the material or improve their skills, the relationship was further considered in the context of the Learning level of the Kirkpatrick model. When those skills were subsequently used to process local food ingredients or develop value-added products, the findings were further interpreted in relation to the Utilization and Access dimensions of the FAO framework.

Following the mapping process, a cross-perspective synthesis was conducted by comparing findings from program documents, trainer perspectives, and participant experiences. This analysis is used to identify patterns of convergence, divergence, and gaps across the three sources of evidence. Synthesis was conducted not only within each individual framework but also across the three frameworks to understand how one stage of the training process relates to another. In this way, the program design could be compared with actual implementation, while participant learning outcomes could be compared with the extent to which skills were applied post-training.

The final stage of the analysis focused on developing higher-level themes that explain the relationships between findings. This process yielded two key mechanisms emerging from the cross-perspective synthesis: the “design–implementation gap,” describing the disparity between the initial design and the need for adaptation during execution; and the “transfer gap,” describing the disparity between successful learning and the practical application of skills. The analysis also identified contextual factors, such as prior experience, access to resources, family support, market opportunities, and socioeconomic conditions that influence the learning transfer process.

Thus, the analytical process progressed from empirical data, initial coding, theme development, interpretation using the three frameworks, cross-perspective synthesis, identification of inter-framework relationships, development of an integrated evaluation framework. This process enabled the construction of the study's final framework based on relationships found within the data, rather than merely placing the ADDIE, Kirkpatrick, and FAO Four Pillars models side-by-side. The results of this synthesis serve as the basis for explaining how instructional design, adaptive implementation, learning outcomes, learning transfer, and contextual conditions collectively shape the training's contribution to community capacity building and the strengthening of sustainable local food systems.

### 1.6 Trustworthiness and data validation

Several strategies were employed to enhance the trustworthiness and credibility of the findings. First, data source triangulation was conducted by comparing information obtained from program documents, trainers, and training participants. These sources provided complementary perspectives on the design, implementation, and outcomes of the training program, allowing the researchers to identify areas of convergence and divergence across sources. In addition, the study employed three complementary conceptual frameworks ADDIE, the Kirkpatrick Model, and the FAO Four Pillars of Food Security to examine the training program from different analytical perspectives. The use of these frameworks should be understood as conceptual triangulation rather than data source triangulation.

Second, member checking was conducted by sharing relevant interpretations and findings with participants and/or key informants to verify the accuracy of the researchers' interpretations and reduce potential misrepresentation of participants' experiences. Third, an audit trail was maintained throughout the research process, including documentation of data collection, coding procedures, theme development, analytical decisions, and revisions to interpretations. Together, these strategies strengthened the credibility, transparency, and dependability of the research findings.

## 2 Result

### 2.1 Strengthening capacity for sustainable local food systems

The training materials extended beyond technical food processing. They included product packaging, business development, and identification of market opportunities, allowing participants to consider how locally available commodities could be transformed into higher-value products. The program therefore reflected an explicit capacity-building orientation in which technical competencies were connected to opportunities for economic activity based on local food resources.

When examined through the FAO Four Pillars of Food Security, the strongest alignment was found with availability and utilization. The availability dimension was addressed indirectly through the program's emphasis on using locally available food commodities as raw materials. The utilization dimension was more directly reflected in the training content, particularly through the development of participants' knowledge and skills to process, package, and diversify local food products. In this sense, the program strengthened participants' capacity to make greater use of food resources that were already available within their local environments.

However, the evidence did not demonstrate an equivalent contribution to the access and stability dimensions. Although the program introduced business development and market opportunities, these components did not establish sustained access to markets, income, or food for participants. Similarly, the documents did not provide evidence that the training itself created mechanisms for maintaining food security outcomes over time. Thus, access and stability remained dependent on conditions beyond the immediate training intervention, including participants' economic resources, market opportunities, and post-training support.

A further limitation was identified in the Analysis stage of the ADDIE process. The program documents did not show a systematic, data-driven assessment of participants' learning needs. Information concerning participant characteristics was obtained primarily through coordination with local governments and program organizers rather than through direct assessment of participants' prior competencies, experiences, and learning needs. As a result, the initial design could not fully anticipate the diversity of participants' social, economic, business, and learning circumstances.

Taken together, these findings indicate that the program had a clear strategic orientation toward local food utilization and community capacity building, particularly in relation to the availability and utilization dimensions of food security. However, weaknesses in needs assessment limited the extent to which the initial instructional design could accommodate the diversity of participants. This limitation subsequently became important during implementation, when trainers had to adapt the planned learning process to actual participant conditions.

### 2.2 Bridging design and implementation

Trainer perspectives revealed that implementation was more dynamic than the instructional design initially anticipated. Although learning objectives and core instructional materials had been established before training as outlined in the final report document for the 2014 training program, trainers reported that the delivery process required continuous adjustment to accommodate differences in participants' prior experiences, motivation, technical abilities, and learning needs.

The diversity of participant experience was particularly important. Some participants had previous experience in food processing or had operated small-scale food businesses, whereas others had little or no experience with commercial food production. Trainers therefore modified their instructional strategies by increasing practical demonstrations, providing more contextual examples, simplifying explanations, and changing the sequence of learning activities.

“*Each participant possesses a different level of cooking knowledge. Some have very limited cooking experience, requiring us to explain the steps slowly and guide them individually to ensure they correctly understand the cooking methods. Consequently, I employ a hands-on teaching approach that combines instruction with practical application, as this is far more effective. Relying too heavily on theory often creates a gap when participants transition to the practical stage, given that theory and practice do not always align perfectly (T1).”*

The quote explicitly explains that participants had different levels of experience, and therefore the trainer had to modify the delivery. Implementation was also affected by operational conditions. Trainers identified limitations in instructional time, the availability of practice materials and facilities, and site-specific circumstances. In response, some instructional materials were simplified, practical sessions were extended, and additional discussions were introduced when participant needs emerged during training.

“*The adaptation process was actually not very difficult, as several participants were already accustomed to attending previous training sessions. We were also familiar with many of them through activities in other organizations, which facilitated smoother interaction. However, implementation did present challenges—specifically regarding practical materials—requiring adjustments based on what was available. Furthermore, the training schedule typically ran from 8:00 AM to 12:00 PM; this duration was not ideal for the cooking practicum. Due to the time constraints, cooking processes requiring sequential steps and precision often could not be executed optimally. My usual strategy was to assist participants who were struggling or unable to keep up with the pace. It was essential to help them catch up and follow the training flow alongside the other participants, often by allowing a little extra time (T2).”*

These adaptations indicated that implementation was not a direct replication of the original instructional design. Rather, trainers acted as mediators between the planned curriculum and the realities of the learning environment. Adaptation therefore functioned as a mechanism for maintaining instructional relevance while simultaneously revealing a discrepancy between what had been planned and what was feasible in practice. This discrepancy constituted the design–implementation gap, which emerged as the first critical transition point in the training process.

This finding is important for the integrated framework because the gap did not necessarily represent program failure. Instead, it demonstrated that the effectiveness of the original design depended partly on trainers' capacity to interpret and adapt the design to the implementation context.

### 2.3 From learning to application: evidence of training transfer

Participant experiences indicated that training outcomes developed differently across the four levels of the Kirkpatrick Model. At the Reaction level, most participants expressed positive perceptions of the training. They considered the learning materials relevant to their everyday needs, found the instructional methods understandable, and valued the hands-on activities because these enabled them to participate directly in the learning process. Many participants also reported greater confidence in applying the local food processing techniques introduced during training.

“*I am truly delighted. Activities like this allow us to update our skills. Until now, we have focused almost exclusively on kitchen operations—what happens behind the oven and stove, how to create delicious products, and how to sell them. Furthermore, the training we received was perfectly aligned with our needs and has genuinely fostered business growth. For instance, the training on equipment usage was highly relevant and has had a very positive impact on us (P1).”*

At the Learning level, nearly all participants reported acquiring new knowledge and technical skills. These included raw-material selection, food-processing techniques, product packaging, and a greater understanding of the economic potential of locally available food resources. Some participants also described a change in how they viewed local commodities, shifting from perceiving them as relatively low-value products to recognizing their potential as sources of income.

“*I have gained new insights into product processing and marketing. While our processes used to be somewhat haphazard, we now have clearer standard operating procedures, which has significantly impacted our production output. Regarding marketing, our reach was previously limited to the local area. We have now started using Instagram, although we haven't yet expanded to other social media platforms. Nevertheless, we have begun to understand the importance of broadening our marketing reach and keeping pace with the evolution of digital platforms (P7).”*

Greater variation emerged at the Behavior level. Some participants applied their newly acquired knowledge by developing new products, improving existing production methods, or expanding household-scale food-processing activities. Other participants, however, reported difficulties in consistently applying the competencies acquired during training because of constraints encountered after the training had ended.

“*The training provided by the Food Security Agency was incredibly helpful to us, particularly regarding the three main topics we covered. First, the online promotion training gave us a better understanding of how to market our products digitally to achieve a wider reach. Second, the financial management training helped us organize our business finances more systematically and keep them separate from personal expenses. Third, the training on product legality and innovation was crucial; having full legal compliance allows us to reassure customers that our products are safe and fit for consumption. All this material is highly relevant and beneficial for the growth of an business like ours. However, some other participants are still facing challenges in applying what was taught, particularly regarding the implementation of online sales via applications, as well as the handling and preparation of business legalities (P8 and P11).”*

The variation was even more evident at the Results level. Some participants reported broader product diversification, increased confidence in managing local food businesses, and increased household income. For others, the benefits remained primarily at the level of knowledge and skills and had not yet developed into sustained economic activity.

“*As the training has only taken place three times, long-term results are not yet apparent—since profound change typically requires time, ongoing guidance, and repeated opportunities to put the knowledge into practice. Nevertheless, those three sessions have established a vital initial foundation across production techniques, marketing, and business management (P11).”*

The differences across these levels indicated that positive reactions and learning outcomes did not automatically lead to consistent behavioral or economic outcomes. Overall, the program demonstrated relatively consistent positive outcomes at the Reaction and Learning levels, whereas outcomes at the Behavior and Results levels varied substantially across participants.

Importantly, the variation in behavioral and economic outcomes was associated with contextual conditions identified across the participant narratives, including participants' previous experience, access to financial and material resources, family and community support, and market opportunities. These findings indicate that training transfer depended not only on what participants learned during the training but also on whether the post-training environment enabled them to apply and sustain those competencies.

### 2.4 An empirical integrated framework for sustainable local food system capacity building

Based on a cross-perspective synthesis of program documents, trainer perspectives, and participant experiences reveals that the research framework originates in the Analysis and Design stages of the ADDIE model (see Figure 1), where training objectives, instructional materials, and delivery strategies are established. However, the analysis indicates that the transition from the design stage to Development and Implementation does not proceed linearly. Variations in participant characteristics and operational conditions necessitate adjustments to the planned instructional process by trainers. This gives rise to the first critical point identified in the synthesis: the gap between design and implementation.

The second critical point emerged when the training outcomes were examined through the Kirkpatrick Model. Although participants reported positive reactions and substantial learning, improvements in knowledge and technical skills were not consistently translated into behavioral changes or sustained economic outcomes. This pattern constituted a transfer gap between learning achievement and practical application.

The synthesis further showed that the two gaps were not independent of the participants' broader environments. Previous experience, access to financial and material resources, family and community support, and market opportunities influenced participants' ability to apply the competencies acquired through training. These contextual conditions helped explain why participants who received the same training did not necessarily demonstrate comparable behavioral or economic outcomes.

When interpreted through the FAO Four Pillars, the strongest contribution of the training was associated with utilization, supported by its emphasis on processing and adding value to locally available food resources. The training also contributed indirectly to availability by strengthening participants' capacity to make productive use of local food resources. In contrast, evidence for direct contributions to access and stability was limited because these dimensions depended on post-training economic conditions, market opportunities, resources, and longer-term institutional support that were beyond the immediate scope of the training intervention.

## 3 Discussions

This study demonstrates that the contribution of the Local Food Processing Training Program to strengthening local food systems cannot be understood solely through successful program delivery or improvements in participants' knowledge and skills. Rather, the findings indicate that training effectiveness depends on whether learning can be transferred and sustained in practice. The central academic finding of this study is therefore the existence of a transfer gap between positive learning outcomes and sustained application, with this gap shaped by the interaction between program design, implementation, and the contextual conditions surrounding participants after training. This interpretation is consistent with the broader understanding that program effectiveness should be examined not only in terms of immediate outputs but also in relation to the context in which those outputs are expected to generate longer-term outcomes (14, 15).

The first part of this process becomes evident in the relationship between program design and implementation. The training was oriented toward strengthening participants' capacity to process and utilize local food resources, which is relevant to efforts to strengthen local food systems. However, the analysis showed that the initial program design did not fully reflect the diversity of participants' prior knowledge, experience, motivation, skills, and business conditions. Consequently, trainers adapted materials, methods, and learning activities during implementation to maintain their relevance to participants' circumstances. This flexibility aligns with adult education principles that recognize the experiences and needs of learners as part of the learning process (16, 17).The findings therefore demonstrate that implementation was not simply a replication of the original design, but an adaptive process in which trainers reconciled planned objectives with actual field conditions. This aligns with the view that the initial analysis process serves as the foundation for subsequent stages, and the quality of information used at that stage determines the extent to which the design can respond to the actual learning context (8, 12, 18).

This finding is consistent with the iterative character of ADDIE (9), but the contribution of the present study does not lie in establishing that ADDIE is iterative. Rather, the study empirically demonstrates how a design–implementation gap emerges when a standardized training design encounters heterogeneous participants and resource constraints, and how trainer adaptations can provide feedback for subsequent program improvement. Thus, the design–implementation gap should not necessarily be interpreted as evidence of program failure. Instead, it reveals where assumptions embedded in the initial design require adjustment when confronted with real-world conditions. This finding also suggests that systematic needs assessment before training is important because greater understanding of participants' starting conditions may reduce unnecessary adaptations during implementation.

The implications of this design–implementation relationship become clearer when the findings are considered through the Kirkpatrick framework. The findings reveal a transfer gap between learning and practice. Although participants generally reported positive reactions to the training and perceived improvements in their knowledge and skills, indicating relatively strong outcomes at the Reaction and Learning levels. However, outcomes at the Behavior and Results levels were more varied. Some participants reported applying what they had learned by developing or improving processed food products, whereas others faced greater difficulties in translating learning into sustained practice.

This study provides a more substantive explanation of variations in training effectiveness than simply determining whether a training program was successful or unsuccessful. Previous research has established that learning transfer is influenced by factors beyond the training itself, including individual characteristics, the work or organizational environment, and available support (5, 6, 19). The findings of this study were also consistent with research in the agricultural and small-enterprise sectors in developing countries, which has shown that increases in knowledge and skills do not always result in adoption or changes in practice when participants face limitations in their supporting environments. For example, a study of smallholder farmers in Uganda showed that participant characteristics, training design, and the work environment were significantly associated with factors influencing the transfer of training outcomes into agricultural practice (20). Similarly, a meta-analysis of 367 agricultural technology adoption models in developing countries showed that access to credit, extension services, education, and organizational membership were consistently associated with technology adoption (21). These findings reinforced the view that technical capacity acquired through training was an important prerequisite but did not, by itself, guarantee transfer into practice.

In the context of local food-processing enterprises in Central Java, the findings of this study illustrated these dynamics more concretely. Participants were able to acquire relevant knowledge and skills during the training, but their ability to apply them depended on access to financial resources, production facilities, raw materials, markets, family and community support, and other socioeconomic conditions. This was consistent with evidence concerning micro and small food-processing enterprises in Indonesia. Rifin (22), for example, showed that the ability of micro and small food-processing industries to expand their markets beyond the local area was associated with business scale, number of workers, education level, access to external financing, and business location. Thus, mastery of processing technology did not automatically eliminate barriers at the commercialization stage. Similarly, a study of micro and small food-processing industries in Indonesia found that access to formal financing was associated with entrepreneurs' asset and educational characteristics, indicating that technical capacity and access to financial resources represented distinct dimensions of business development (23).

Such dynamics help explain the barriers to markets or capital that participants in this study may face after mastering food-processing technologies. At the micro and small enterprises level, new technologies had to be translated into production activities that required investment, consistent raw materials, proper facilities, product packaging and quality and marketing channels that allowed products to reach consumers. In Indonesia, evidence suggested that financing constraints were an important barrier to the growth of micro and small food-processing enterprises, including cassava-processing businesses, while access to financing could be associated with business performance (24). As a whole, research on Indonesian MSMEs also identified financing, logistics, regulatory complexity and market access as interrelated barriers to business development.

This dynamic was also evident in an intervention study involving small-scale producers in the Philippines. Hossain et al. (25) found that a combination of training, credit, and infrastructure increased market participation and income, while the benefits of the intervention were more limited in more remote areas. This finding was important for interpreting the present study because it indicated that training could not always function independently as a mechanism for generating economic change; rather, production support needed to be connected to conditions that enabled producers to access markets and make use of the knowledge and skills gained through training. In other words, when training increased technical capacity without simultaneously strengthening access to resources and supportive environments, some of the benefits of learning could remain at the *learning* level and not be fully translated into *behavior* or *results*.

Within the local food-system context of Central Java, these conditions indicated that learning transfer needed to be understood as a process occurring within an enterprise ecosystem rather than as a direct relationship between training and behavioral change. Participants operated in an environment in which production capacity, financing, raw materials, marketing networks, and institutional support were interconnected. Therefore, participants could possess the ability to perform new practices without necessarily having the conditions required to sustain those practices over time. The resulting gap was not primarily between “knowing” and “not knowing,” but between “being able to perform” and “being able to sustain the application under real-world conditions.” Within the *New World Kirkpatrick Model*, the application of learning after training was an important component in determining training value because learning generated benefits only when participants were able to apply it and the environment provided the support necessary to sustain the resulting behaviors (26).

Thus, the gap that arises was not fundamentally a failure of learning; rather, it reflected a difference between the capabilities successfully developed through training and the systemic conditions available to activate and sustain those capabilities. This finding extended the understanding of training effectiveness in local food-enterprise contexts by showing that program evaluation needed to move beyond asking whether participants acquired knowledge and skills. Evaluation also needed to consider whether the economic, market, institutional, and social environments surrounding participants provided conditions that enabled learning to be transferred into enterprise practices. From this perspective, it represented a central finding that connected the *learning* level with *behavior* and *results*, while also indicating that the success of capacity-building programs depended not only on the quality of the learning intervention but also on the capacity of the surrounding system to enable the resulting learning to be used and sustained.

This finding also helps clarify the relationship between training and the broader food system. The training made its most direct contribution to the FAO Utilization pillar by strengthening participants' capacity to process and develop local food products, while also having implications for Availability through greater use and value addition of local food resources. However, the evidence for Access and Stability was less direct because these dimensions depend on broader economic, market, institutional, and infrastructural conditions. The findings therefore suggest that training can strengthen an important component of local food-system capacity but cannot independently produce all dimensions of food-security improvement.

Within the Utilization pillar, diversification of processed food products provides a potential pathway through which training-generated capabilities may contribute to local food-system outcomes. In theory, processing can transform locally available commodities into different food forms and products, thereby increasing the variety of foods potentially available for household and community consumption. Processing may also extend the usability or storage period of certain commodities, while the development of products from diverse local food resources can expand the range of food choices available within communities. However, this pathway should not be interpreted as evidence that the training directly improved dietary diversity. The present study did not directly measure dietary diversity, dietary intake, or nutritional status. Rather, the findings indicate a potential pathway from increased local food-processing capacity to greater variety and utilization of local food resources, which should be tested directly in future research.

This distinction is important because increased product diversification does not automatically result in greater dietary diversity. Whether diversified processed foods contribute to dietary diversity depends on their affordability, accessibility, nutritional composition, cultural acceptability, availability, and actual consumption by households. The FAO framework therefore helps situate the training within a broader food-system context and prevents the contribution of increased processing capacity from being interpreted as a direct nutritional outcome (1–3).

Overall, these findings demonstrated why integrating ADDIE, Kirkpatrick, and the Four Pillars of FAO was useful for evaluating this type of training program. The contribution of this integration did not lie in presenting these established frameworks as a new theory. Rather, their combined application enabled the study to trace the pathway from program design and implementation, through learning and transfer, to its potential contribution to the food system. ADDIE helped identify how the program was designed and adapted; Kirkpatrick revealed the points at which positive learning outcomes became less consistent at the Behavior and Results levels; and the Four Pillars of FAO situated these outcomes within the broader dimensions of food security. By connecting these three perspectives, the study made a theoretical contribution by conceptualizing capacity-building program evaluation as a staged and conditional pathway rather than as a direct relationship between training and food-system outcomes. Within this conceptualization, success at one stage did not automatically ensure success at the subsequent stage because learning transfer depended on conditions that enabled participants to apply and sustain their learning in practice.

From this integrated perspective, gaps can arise during the training process and afterwards, when participants attempt to apply what they have learned. This highlights the need to align the program design with the participants' actual conditions. This transfer gap represented the central finding of the study because it connected training effectiveness with the broader conditions required for sustainable food-system change. In other words, the study indicated that strengthening learning was important but insufficient: the contribution of training depended on whether the post-training environment enabled participants to translate their learning into sustainable practice. Thus, the integration of ADDIE, Kirkpatrick, and the Four Pillars of FAO expanded the focus of evaluation from “whether participants learned” to “how learning was transferred, what conditions enabled or constrained that transfer, and the extent to which the resulting outcomes had the potential to contribute to the food system.” This shift was important for evaluating capacity-building programs because it allowed the sources of program limitations to be distinguished between design and implementation issues on the one hand and post-training transfer barriers and systemic conditions on the other.

This interpretation had important implications for how public training programs were designed and evaluated. Needs assessments needed to be strengthened before training to identify participants' prior knowledge, experience, motivation, business characteristics, and available resources. At the same time, adaptations made by trainers during implementation needed to be documented and considered in refining subsequent program cycles. Most importantly, evaluation needed to extend beyond participant satisfaction and immediate learning outcomes to examine whether participants had the resources, opportunities, institutional support, and market conditions necessary to transfer their learning into actual practice. Such an approach could help program managers distinguish between limitations originating from training design and constraints arising from the post-training environment.

The findings also indicated that training programs intended to support food-system development should not be implemented as stand-alone interventions. Knowledge and skills provided an important foundation, but sustained application could require complementary support in the form of financing, equipment, market access, mentoring, institutional coordination, and post-training assistance. This was particularly relevant to the Access and Stability dimensions of the FAO framework, which could not be addressed through training alone. Therefore, training effectiveness needed to be assessed within the broader capacity-development system in which the intervention operated, rather than being evaluated solely on the basis of activities completed during the training period.

Future research needed to extend the examination of the transfer gap identified in this study by investigating the transition from learning to sustainable practice over time. Longitudinal research could determine whether the gap between learning and behavior narrowed or widened after training and could identify the conditions that supported sustained application. Further comparative research across regions and training programs could also look at whether this phenomenon was specific to the Central Java context or indicative of broader patterns in public training in agriculture and food-related sectors. Future research could also directly measure dietary diversity, food consumption and nutritional outcomes to investigate whether diversification of local processed food products resulted in actual improvements in the Utilization pillar. Such research could go beyond identifying the potential pathway observed in this study and provide empirical evidence of longer-term effects on the food system.

Overall, the study found that the effectiveness of local food-processing training could not be judged from positive participant response or acquisition of new knowledge and skills. Theoretically, this finding indicated that the relationship between capacity building and food-system change needed to be understood as a staged process that depended on transfer conditions rather than as a linear relationship from training intervention to food-system outcomes. The ADDIE, Kirkpatrick, and Four Pillars of FAO frameworks collectively provided a means of tracing this process from program design and implementation to learning, transfer, and potential contribution to the food system. Thus, the study conceptualized training not as an isolated short-term learning intervention but as one component of a broader capacity-development system, in which the ability to transfer and sustain learning ultimately determined the extent to which training could contribute to local food-system change.

The cross-perspective synthesis also represents an important boundary of the present study. By integrating program documents, trainer perspectives, and participant experiences, the study provides a broader understanding of how design, implementation, learning transfer, and contextual conditions interact. However, these perspectives represent different stages and levels of the program rather than a longitudinal follow-up of the same participants. Therefore, the identified relationships should be interpreted as a cross-perspective synthesis of evidence rather than as a directly observed causal sequence. This distinction also differentiates the present study from Prasetyo et al. (12), which focused specifically on the Analysis and Design phases of the program.

## 4 Limitations of the study

This study has several limitations that should be considered when interpreting its findings. First, the qualitative design and purposive sampling of 15 participants were intended to generate in-depth accounts of participants' experiences rather than statistically representative estimates of programme effectiveness. The findings therefore provide contextual insights into learning transfer and the conditions surrounding it, but they should not be generalized statistically to all participants in local food-processing training programmes or to all communities in Central Java.

Second, although the study incorporated programme documents, trainer perspectives, and participant experiences to support data-source triangulation, the study did not longitudinally observe participants' practices over an extended period. The identified transfer gap should therefore be interpreted as an empirically grounded account of participants' experiences and reported conditions rather than as a direct longitudinal measurement of the persistence of behavioral change.

Third, the study did not directly measure broader food-system outcomes. In particular, it did not quantitatively assess changes in household dietary diversity, food consumption, nutritional status, household food expenditure, business income or profitability, market share, or changes in the availability, access, and stability of food at the community level. Therefore, the relationship between training outcomes and the Four Pillars of FAO should be interpreted as a potential contribution pathway rather than evidence of direct changes in food-security outcomes.

Finally, the study examined a specific local food-processing training programme within the institutional and socio-economic context of Central Java. The barriers identified in relation to capital, markets, production facilities, raw materials, and social and institutional support may operate differently in other regions or agricultural and MSME contexts.

## 5 Conclusions

The study revealed that the contribution of training programs to strengthening local food systems cannot be understood solely by successful program delivery or participants' improved skills. It needed to be seen as a process linking program design, implementation, learning transfer, and the contextual conditions affecting the application of training outcomes. Combining analyses of program documents, trainer perspectives, and participant experiences, the study identified training as a first step in building community capacity to use local food resources and develop food-based economic activities. However, the capacity built through training did not automatically translate into behavioral change or sustained economic impacts. Instead, success depended on the interrelatedness of the training stages and on a supportive environment that enabled participants to apply the competencies they learned.

These findings advanced training program evaluation by showing that integrating the ADDIE model, the Kirkpatrick Evaluation Model, and the FAO's Four Pillars of Food Security provided a more comprehensive understanding of how training strengthened local food systems. This holistic approach went beyond simply measuring training outcomes to describing the actual processes that connected planning, execution, learning, and program outcomes in real-world situations. The evaluation framework of this research also identified the design-implementation gap and the transfer gap as two critical points that need to be addressed when designing training-based interventions, since both determine the extent to which individual capacity building could evolve into broader changes in local food entrepreneurship and sustainable food systems.The implications of this study extended beyond the context of the Local Food Processing Training Program in Central Java. For program developers, the findings emphasized the need for investment in training that goes beyond curriculum design and program implementation to include strengthening needs assessments, establishing post-training support mechanisms, and considering the contextual factors that affected participants' ability to apply what they had learned. This study provided an assessment framework for academics and researchers that could be modified and applied to assess different capacity-building programs to support food system transformation, especially in developing countries with similar challenges in connecting learning to socio-economic change.

Future research should use a longitudinal mixed methods design to determine if capacity improvements immediately following training can be maintained and translated into long-term changes in behavior, economics and food security. In particular, data collection could be carried out at different time points, such as pre-training, immediately after training and a number of points in time for follow-up, which allows a more systematic observation of change across the levels of learning, behavior and results. The approach can also combine qualitative data on participant experiences and contextual factors with quantitative indicators on income changes, business development and sustainability of food processing practices. To better measure economic impact, future research needs to consider including a return on investment (ROI) index, which measures the economic benefits achieved against program costs and post-training support. Changes in household food security status could also be measured simultaneously by the Household Food Insecurity Access Scale (HFIAS) to see whether participants' capacity building and economic activities are associated with changes in household-level food access. Thus, future longitudinal research can provide stronger evidence on whether and how training shifts from an individual level capacity building intervention to a wider contribution to local food entrepreneurship, household wellbeing and a safe, resilient and sustainable food system.

## Data Availability

The raw data supporting the conclusions of this article will be made available by the authors, without undue reservation.

## References

[1] Food and Agriculture Organization (FAO). An Introduction to the Basic Concepts of Food Security (2008). Available online at: https://www.fao.org/4/al936e/al936e00.pdf (Accessed July 05, 2026).

[2] Prasetyo AS Dooley KE Widyastuti K Firdauzi A. Comparing national food security policies in Indonesia and Thailand: a systematic meta-synthesis approach. Front Sustain Food Syst. (2025) 9:1676641. doi: 10.3389/fsufs.2025.1676641

[3] Food and Agriculture Organization (FAO). The Future of Food and Agriculture: Trends and Challenges (2017). Available online at: https://openknowledge.fao.org/server/api/core/bitstreams/2e90c833-8e84-46f2-a675-ea2d7afa4e24/content (Accessed July 05, 2026).

[4] High High Level Panel of Experts on Food Security and Nutrition (HLPE). Food Security and Nutrition: Building a Global Narrative Towards 2030 (2020). Available online at: https://openknowledge.fao.org/server/api/core/bitstreams/8357b6eb-8010-4254-814a-1493faaf4a93/content (Accessed July 05, 2026).

[5] Baldwin TT Ford JK. Transfer of training: a review and directions for future research. Pers Psychol. (1988) 41:63–105. doi: 10.1111/j.1744-6570.1988.tb00632.x

[6] Burke LA Hutchins HM. Training transfer: an integrative literature review. Hum Resour Dev Rev. (2007) 6:263–96. doi: 10.1177/1534484307303035

[7] Grossman R Salas E. The transfer of training: what really matters. Int J Train Dev. (2011) 15:103–20. doi: 10.1111/j.1468-2419.2011.00373.x

[8] Branch RM. Instructional Design: The ADDIE Approach. New York, NY: Springer (2009).

[9] Molenda M. In search of the elusive ADDIE model. Performance Improvement. (2015) 42:34–6. doi: 10.1002/pfi.4930420508

[10] Kirkpatrick DL Kirkpatrick JD. Implementing the Four Levels: A Practical Guide for Effective Evaluation of Training Programs. San Francisco, CA: Berrett-Koehler (2007).

[11] Kirkpatrick J Kirkpatrick WK. The kirkpatrick model: past, present and future. Chief Learn Officer. (2009) 8:20–55. Available online at: https://www.cedma-europe.org/newsletter%20articles/Clomedia/The%20Kirkpatrick%20Model%20-%20Past,%20Present%20and%20Future%20(Nov%2009).pdf (Accessed July 5, 2026).

[12] Prasetyo AS Dooley KE Briers GE Locke DC Shehane M. Evaluation of local food processing training design based on ADDIE and FAO food security pillars in central Java. Agrisocionomics. (2026) 10:617–31. doi: 10.14710/agrisocionomics.v10i2.31905

[13] Yin RK. Case Study Research: Design and Methods. 5th ed. Thousand Oaks, CA: Sage (2014).

[14] Patton MQ. Essentials of Utilization-Focused Evaluation. Thousand Oaks, CA: Sage (2012).

[15] Rossi PH Lipsey MW Henry GT. Evaluation: A Systematic Approach. 8th ed. Thousand Oaks, CA: Sage (2019).

[16] Knowles MS Holton EF III Swanson RA. The Adult Learner: The Definitive Classic in Adult Education and Human Resource Development. 8th ed. New York, NY: Routledge (2015).

[17] Merriam SB Bierema LL. Adult Learning: Linking Theory and Practice. San Francisco, CA: Jossey-Bass (2014).

[18] Morrison GR Ross SM Morrison JR Kalman HK. Designing Effective Instruction. 8th edn. Hoboken, NJ: Wiley (2019).

[19] Salas E Tannenbaum SI Kraiger K Smith-Jentsch KA. The science of training and development in organizations: what matters in practice. Psychol Sci Public Interest. (2012) 13:74–101. doi: 10.1177/1529100612436661 26173283

[20] Twase I Miiro RF Matsiko F Ndaula S Ssamula M. Contextual issues influencing training transfer system factors among smallholder farmers in central Uganda. African J Rural Dev. (2021) 6:91–103. Available online at: https://afjrdev.org/index.php/jos/article/view/303

[21] Ruzzante S Labarta R Bilton A. Adoption of agricultural technology in the developing world: a meta-analysis of the empirical literature. World Dev. (2021) 146:105599. doi: 10.1016/j.worlddev.2021.105599 PMC8479612 34621923

[22] Rifin A. Determinants of micro and small enterprise food industry market expansion in Indonesia. Indonesian J Business Entrepreneurship. (2019) 5:215. doi: 10.17358/ijbe.5.3.215

[23] Tambunan TT. MSMEs and access to financing in a developing economy: the Indonesian experience. In: Financial Entrepreneurship for Economic Growth in Emerging Nations. Hershey, PA: IGI Global Scientific Publishing (2018). p. 148–72.

[24] Taslim L Rifin A Jahroh S. Pengaruh pembiayaan terhadap kinerja usaha mikro dan kecil olahan ubi kayu di Indonesia. J Agribisnis Indonesia. (2020) 8:33–42. doi: 10.29244/jai.2020.8.1.33-42

[25] Hossain M Mendiratta V Mabiso A Songsermsawas T. Training, credit, and infrastructure for improving market access among small-scale producers in the Philippines. Food Policy. (2025) 132:102853. doi: 10.1016/j.foodpol.2025.102853

[26] Kirkpatrick J Kirkpatrick WK. An introduction to the New World Kirkpatrick^®^ *Model* (2021). Available online at: https://www.fieldepiinaction.com/s/Kirkpatrick-New-World-Model.pdf (Accessed August 29, 2026).

